# Rational Suicide, Euthanasia, and the Very Old: Two Case Reports

**DOI:** 10.1155/2016/4242064

**Published:** 2016-10-19

**Authors:** Anne Pamela Frances Wand, Carmelle Peisah, Brian Draper, Carolyn Jones, Henry Brodaty

**Affiliations:** ^1^Older Persons' Mental Health Service, St George Hospital, Kogarah, NSW, Australia; ^2^Academic Department for Aged Care Psychiatry, Eastern Suburbs Mental Health Service, Randwick, NSW, Australia; ^3^School of Psychiatry, University of New South Wales, Sydney, NSW, Australia; ^4^Discipline of Psychiatry, Sydney Medical School, University of Sydney, Sydney, NSW, Australia; ^5^Dementia Collaborative Research Centre and Centre for Healthy Brain Ageing, University of New South Wales, Sydney, NSW, Australia

## Abstract

Suicide amongst the very old is an important public health issue. Little is known about why older people may express a wish to die or request euthanasia and how such thoughts may intersect with suicide attempts. Palliative care models promote best care as holistic and relieving suffering without hastening death in severely ill patients; but what of those old people who are tired of living and may have chronic symptoms, disability, and reduced quality of life? Two cases of older people who attempted suicide but expressed a preference for euthanasia were it legal are presented in order to illustrate the complexity underlying such requests. The absence of a mood or anxiety disorder underpinning their wishes to die further emphasises the importance of understanding the individual's narrative and the role of a formulation in guiding broad biopsychosocial approaches to management.

## 1. Introduction 

The population is ageing globally, particularly the proportion of those who are very old, and it is this group who appear most vulnerable to dying by suicide in most countries [1]. Epidemiological studies of community dwelling older adults have estimated that the wish to die has a point or recent (within the past two weeks) prevalence of 2–6% [2–4]. Suicidal ideation occurs in a smaller proportion. Various factors are associated with the wish to die, including depressive symptoms (major depression only occurs in a minority), social isolation, anxiety, pain, disability, and institutionalisation.

It has been hypothesised that death wishes and suicidal ideation might be “understandable” in some contexts and that such thoughts could emerge as positive solutions to regain control or provide release after an accumulation of life events or problems of ageing [2, 5]. Suicides in older people without psychiatric disorder that occur in these circumstances have been classified in some studies as “rational suicide” [6]. Rational suicide has been defined as occurring when a person is able to reason, possess sufficient information, have a realistic worldview, and act (to end their own life) according to their own essential interests [7]. Rationality is probably dimensional rather than dichotomous [8]. For example, having depression does not imply that a person's choice is irrational and, conversely, rationality cannot be assumed when depression or mental illness is absent. However, mental illness may influence decision making and, even in those who have previously attempted suicide whose acute depressive illness has resolved, there may be persistent impairment in decision-making capacity [9]. Compared to requests for euthanasia in people with a terminal illness, there is additional complexity in evaluating the same request in someone with a mental illness. It has been argued that the request for euthanasia in this context may be a symptom of the illness itself, the prognosis of some mental illnesses may be less certain, the impact of the illness on decision making may be more variable, and there are no objective indicators for predicting response to treatment [9–11]. Additionally, the determination of rationality is subjective and influenced by one's personal view of ageing, older people, and the psychological effects of disability and chronic disease [8].

The wish to die has been explored qualitatively in the Netherlands where euthanasia is legalised [5] but not directly in Australia where euthanasia is not legal. Although suspected, it is unknown whether people expressing a wish to die differ from those who attempt suicide [5] or if there is a continuum from the wish to die to suicide attempts and completed suicide [3]. Moreover, these issues have not been explored qualitatively in the very old. We present two case reports of older people who attempted suicide and had requested euthanasia, in order to highlight the individual at the centre of the request, the complexity of the contextual factors contributing to wishes to die, and how these factors may guide personalised interventions to reduce distress. Both patients provided consent for publication.

## 2. Case Presentations 

### 2.1. Case  1 

Mrs. C, aged 88, was the sole carer of her frail older husband. She was a retired typist and then home maker. Her two daughters lived overseas. Mrs. C was admitted under geriatric medicine after a planned insulin overdose with intent to die. An aged care psychiatrist was asked to assess her on the medical ward. She revealed she had left a farewell note to her family, did not expect to be found, and wished she had died. Mrs. C had acute chronic back pain for which she had received various treatments without relief. Other medical comorbidities included diabetes, ischaemic heart disease, hypertension, osteoarthritis, and supraventricular tachycardia. Her husband relied upon her to maintain their household and to assist him with personal care, although her pain precluded this. She had become increasingly worried about her husband's mounting ill health and disability reaching a point where she would be unable to help him. She was worried about becoming a burden and dependent on others and the prospect of residential care, about which they were both strongly opposed. In recent weeks the couple's lives revolved around medical ailments and appointments. She had become more disheartened with each of her husband's recent unsuccessful surgical procedures. Pain and physical disability had reduced their mobility, limiting their ability to socialise.

Mrs. C only spoke of herself with reference to her husband and the help or hindrance she could provide him rather than seeing herself as an individual deserving of care and quality of life. Unable to fulfil her role as his carer, she had little reason to live. The couple had long discussed euthanasia as an option if ever they developed intractable symptoms and lost their independence. Mrs. C was frustrated euthanasia was not available to her. She had subsyndromal depressive symptoms, including a sense of hopelessness and helplessness. She was not anhedonic and denied reduced talkativeness or social withdrawal, self-pity, or pervasive pessimism, symptoms which are more specific for depression as opposed to somatic features in the context of medical illness [12]. She had been sleeping more to escape the pain. There was no past psychiatric history in her or her family. Her husband had not observed any change in her mood or other signs of depression or anxiety. No neuroimaging was performed.

On examination, Mrs. C was well groomed and maintained good eye contact. Rapport was tenuous and she appeared uncomfortable during the assessment. Mrs. C had difficulty describing her emotions but denied feeling depressed. Her affect appeared bland and detached, although reactive and with a full range. Her thought form was normal. She described feeling helpless and hopeless and still wished to die but did not have any specific plans to end her life. There were no anxious cognitions or psychotic symptoms. Mrs. C's cognition was normal on Mini Mental State Examination, as she scored 29/30 [13]. Aside from assistance with pain relief she did not identify any other problems or need for treatment.

The consulting psychiatrist noted the depressive cognitions but a lack of other symptoms necessary to reach a diagnosis of major depression. Minor depression was a differential diagnosis. The suicide attempt occurred in the context of acute chronic pain (associated with a sense of helplessness and hopelessness), disability, reduced mobility, and concern about her husband's deteriorating health. Her cognisance of their gradual loss of independence and her inability to continue her caregiver role contributed to the wish to die. She had few coping strategies or external supports to call upon to assist her.

The geriatrics team replaced Mrs. C's panadeine forte (paracetamol and codeine) with pregabalin, paracetamol, and oxycodone/naloxone, which led to minor improvement in her pain. She participated in sessions with the ward physiotherapists and was independent in activities of daily living, including mobility. She was offered but declined additional services to assist with domestic duties and care for her husband. Mrs. C was transferred to a private hospital for further optimisation of analgesia and declined psychiatric follow-up. The team discussed a step-wise plan for management of acute pain at home with Mrs. C. She was discharged home with her husband, with some increased domestic support at home and geriatric follow-up.

### 2.2. Case  2

Mr. B, aged 89, was a retired businessman and widower of 30 years who lived alone. He had two children who lived interstate. He was referred by his case manager for an urgent assessment due to disclosure of recent suicide attempts and an ongoing wish to die. He had been discharged from the Older Persons' Mental Health unit a few weeks prior, where he had been admitted for suicidal ideation disclosed to a mental health hotline. During the two-week admission his psychiatrist concluded that he did not have a major depressive illness, but he was found to misuse alcohol and had an eighteen-month history of cognitive and functional decline. Cognitive impairment was noted on the Montreal Cognitive Assessment Test [14], with a score of 23/30. Points were lost for short-term memory, executive function, language, and orientation. Detailed neuropsychological assessment revealed significant impairment in executive function (especially inhibitory control, impulsivity, poor organisational skills, and impaired cognitive flexibility) and memory (new learning). He was advised not to drive and the Roads and Maritime Services (RMS, the State Drivers' Licencing Authority) was notified about his cognitive impairment and the need to review his licence. There were no acute medical issues identified, but his history was significant for previous myocardial infarction, coronary angioplasty, coronary artery bypass graft, mitral valve replacement, hypertension, diabetes, glaucoma, ulcerative oesophagitis, and an unrepaired inguinal hernia. Neuroimaging revealed moderate dilatation of the ventricles and subarachnoid cisterns and periventricular white matter hypoattenuation reported to be consistent with chronic small vessel ischaemia. Mr. B had declined follow-up with the drug health team and was precontemplative regarding viewing his alcohol use as problematic or contributory to his suicidality. Mr. B had rejected the option of living in a residential aged care facility primarily as he prioritised his independence and was fearful of becoming fully dependent as his mother did from the ages 92–100. He described her as existing like a “pot plant,” possibly with underlying dementia. He also wanted to avoid causing grief and burden to his family who would feel obligated to visit him, as he had felt visiting his mother. A secondary concern was the cost of residential care, which he saw as exorbitant. He did not want to diminish the value of his estate, which he wanted to pass on to his children. His suicidal ideation self-resolved without any pharmacological treatment and he had been discharged with psychogeriatric follow-up and a weekly cleaning service.

Mr. B was assessed by an aged care psychiatrist at his home with his mental health case manager. He reported initially settling in well at home following discharge and had driven short distances to run errands. He received a letter from the RMS informing him that his drivers' licence had been cancelled. This, along with initial insomnia, led to resumption of drinking alcohol, three to four standard units per night. Without the ability to drive, he described lacking any usefulness or purpose in life as he could no longer babysit a friend's children or access the shops independently. There was no change in appetite or energy levels. He had come to feel old with the gradual development of difficulty walking, balance problems, and reduced exercise tolerance. He reflected that he would not achieve anything more in life. He was worried about becoming a burden on his children. Mr. B continued to enjoy social outings with his sister and visits from his neighbours, but he had found it hard to organise his day and often wandered his apartment wondering what to do.

He lamented that euthanasia was not legal and had therefore concluded the only solution was to end his life. A week earlier he had tried to end his life by covering his head with a plastic bag but aborted the attempt as the sensation of suffocation was too distressing, reminiscent of a near-drowning experience in childhood, and “the body fought it.” A few days prior to the urgent assessment he had written an explanatory farewell note to his children, purchased extra-large garbage bags to include his head and torso, and repeated the suicide attempt. A similar involuntary response led to abandoning the attempt and he subsequently tore up the note. Mr. B had spent much time contemplating other ways of ending his life, including stabbing an artery, but was unsure how to go about it and did not want to try and fail.

Mr. B was engaging and developed a warm rapport but was easily distracted by items in his home. He walked holding onto furniture. He was spontaneous in conversation. He described his mood as bored and lost “like I'm floating around” although his affect was euthymic, reactive, and sometimes jovial. He was thought disordered; in particular he was overinclusive and circumstantial. He described himself as useless and lacking purpose given his age and loss of independence and felt hopeless. He had ruminating thoughts about how he could end his life. There were no delusions or perceptual disturbances. Although not formally reassessed cognitively, he was perseverative, misplaced items during the interview demonstrating poor short-term memory, and was dyspraxic. He maintained that he should be allowed to choose to die and was sceptical his situation might improve or there could be scope to find quality or meaning in his life.

It was again concluded that he did not have a depressive illness but rather cognitive impairment which, combined with his loss of independence, his role, and identity, had led to a loss of purpose and boredom. The alcohol was considered an important contributing factor which exacerbated his executive dysfunction, leading to even more impulsivity and disinhibition. Understimulation and the lack of skills to structure his day were likely factors leading to recurrent suicide attempts.

Mr. B returned to the mental health unit for further evaluation and exploration of management options which might reduce his wish to die. In hospital he revealed a further recent suicide attempt at home, by carbon monoxide poisoning. He had bought a hose and tested it out. He was treated with parenteral thiamine and placed on an Alcohol Withdrawal Scale and protocol. A capacity assessment was recommended to determine his ability to make decisions about his type of accommodation and the use of external services. His sister and children were contacted regarding his presentation.

On the ward Mr. B interacted with other patients, enjoyed watching the football, and ate well, and his sleep improved. No pharmacotherapy was initiated. Following a series of discussions with Mr. B and his family he decided that he would move into a residential aged care facility in another state to be close to his son. He identified having people to socialise with and a daily routine as positive aspects of this choice and that this would reduce some of his children's worries about him. Mr. B was optimistic about having an active role in his young grandchildren's lives. He was reassured about the financial implications of this decision and was able to participate in making decisions about the choice of facility and how he would fund this. He was pleased that he would be assisted to organise the move as he found the task daunting.

## 3. Discussion 

These cases illustrate that requests for euthanasia may occur in older people in the absence of a significant mood disorder. They highlight the unique interplay of biopsychosocial factors, such as cognitive impairment (including emerging dementia), low mood, substance misuse, pain, disability, and social impoverishment, which may underlie requests for euthanasia in very old people. Some of these individuals may be chronically bored, restricted in activity, and have little or no avenues to experience any pleasure in life, such that they are “waiting to die.”

Physicians in the Netherlands estimated that approximately 17% of requests for euthanasia came from patients who were tired of living [15], a request which does not meet the country's legal requirement for euthanasia of a “medical cause.” Although both patients had attempted suicide neither was thought to have major depression. Both were tired of life, struggled with the prospect of becoming dependent upon others, were scared of the prospect of institutional care, and could see no solution other than to end their lives. The cases also highlight the significance of perceived loss of role and purpose in late life. For Mrs. C the mounting disability and pain of her chronic medical conditions, loss of role as a carer, and illness in her frail older husband were important contributing factors, whereas in Mr. B's case his own negative views of ageing, perceived lack of usefulness as an older person, loss of his driving licence and independence, and his executive dysfunction limiting his ability to plan and structure his day and life had led him to conclude death was the only option. Others have similarly identified that some suicides in older people stem from the individual's perception that there is no other way out of an untenable situation [6].

Lindesay [16] suggested that elderly suicides are often explained as rational choices, especially if the person has physical illnesses, which may reflect ageism and therapeutic nihilism. Ageism, that is, stereotypes and assumptions about older people made purely on the basis of their age, may affect the ability of clinicians to detect the psychopathology that frequently underlies suicide in late life [8]. This may occur if depression is deemed understandable in the context of an older person's health and living circumstances if the potential reversibility of their depressive illness is underestimated or if suicidal thoughts are validated although previously unrecognised alternatives exist [8]. Indeed Mr. B himself had negative views of ageing as a state of being useless to society and unproductive compared to his working years. Arguably, in Mrs. C's case, therapeutic nihilism or ageism could have been present as clinicians initially took a less aggressive approach to pain management. The impact of such attitudes in staff highlights an opportunity to intervene with clinicians to improve the care of older patients before they reach the conclusion that nothing can be done to help their distress. In relation to requests for euthanasia from people with mental illness, it has been suggested that considering such requests may add to clinician pessimism and stigma about mental illness by reinforcing poor expectations of treatment effectiveness and ultimately resulting in clinicians giving up on treating some patients and reducing the impetus for improving psychiatric services [9, 11].

Medical paternalism is another potential form of ageism, where coercive life-prolonging measures may be implemented without consideration of the person's autonomy. With Mr. B, had he not had capacity to make a decision about his living arrangements, it is possible that guardianship and an accommodation order may have been sought by the team.

There are numerous studies elucidating population-level risk factors for suicidal ideation or completion in older people (see, e.g., [4, 17]). However, there is little formal literature to guide clinicians in how to help older people without major psychiatric illness who wish to die [18]. Although this lack of guidance is problematic for all jurisdictions, it is perhaps particularly relevant in settings where euthanasia is legal. For example, although depression was the most common diagnosis in a recent review of Dutch cases of euthanasia for people with psychiatric disorders, significant comorbidity was found with personality disorders, medical illness and functional impairment, loneliness, and social isolation [10]. Refusals of treatment were also common, confounding evaluation of the futility requirement [10]. The cases illustrate that interventions specific to the individual's needs may relieve suffering. In Rurup et al.'s [5] explanatory framework for how a wish to die may develop, the balance between wishes to die and to live derives from a complex interplay of life events, ageing, and illness combined with coping strategies, personality, social support, and issues of control. Clinicians need to have an open mind when evaluating psychological symptoms and comorbidity and treatability in the context of death wishes, emphasising that the wish to die may reflect a valid assessment of quality of life and means of regaining control [5].

The wish to die may be a passive thought quite distinct from the desire for euthanasia. A detailed narrative formulating the derivation and meaning of the wish to die may provide the person, their family, and treating team with targets for intervention. Accordingly, requests for euthanasia in the terminally ill have been considered “cries for help” from patients who are suffering or family members who have difficulty coping with the illness, which often resolve when a skilled clinician explores and addresses the real issues at hand [19] or in the depressed elderly when depression is treated [20]. Whilst in these two cases the recommendations to address their risk factors were not fully accepted by the individuals for various reasons, there is evidence that when treated for distressing symptoms or psychosocial problems some older people withdraw their request for euthanasia [18]. However, if a wish to die is prevalent and persistent in some [20], it may be an acceptable residual symptom if all treatable factors have been addressed.

Clinicians have several options for addressing the wish to die in their older patients. Crafting an advance care directive may give relief for some to know that their life will not be necessarily prolonged and provide an opportunity to exert some control over their lives. For others, exploration of spiritual issues and facilitating pastoral care may be of value [21]. In practice, it may be the empathic, ongoing care and support provided by primary care clinicians that people want [21]. Children may find the development of dependency and proximity of mortality in their parent confronting. Long-held conflicts and disputes may reemerge as families grapple with these issues. Acknowledging and supporting both patients and their families during this time are necessary. As stress and social disconnection have also been associated with suicidal thoughts, social interventions to address these issues have been suggested too [4]. For example, a telephone welfare monitoring, emotional support, and emergency alarm service for older people resulted in fewer suicide deaths than expected [22]. For Mr. B, moving to an aged care facility provided structure as well as ready access to social interactions unavailable to him at home.

There must be a balance between therapeutic nihilism and acceptance of a phenomenon associated with increased longevity. Exploring the unique narrative for the older person presenting with a wish to die or following a suicide attempt provides foci for tailoring recovery based interventions, which go beyond merely assessing for a mood disorder, which is often not present. The request for euthanasia should never be considered “understandable” merely because a person is aged. By addressing the range of issues contributing to the individual's wish to die, distress and suffering may be reduced and requests for euthanasia withdrawn.
